# Action Recognition in Basketball with Inertial Measurement Unit-Supported Vest

**DOI:** 10.3390/s25020563

**Published:** 2025-01-19

**Authors:** Hamza Sonalcan, Enes Bilen, Bahar Ateş, Ahmet Çağdaş Seçkin

**Affiliations:** 1 Computer Engineering Department, Engineering Faculty, Aydın Adnan Menderes University, Aydın 09100, Türkiye; hamzasonalcan@gmail.com (H.S.); enesbilen80@gmail.com (E.B.); 2 Faculty of Sport Science, Uşak University, Uşak 64100, Türkiye; bahar.ates@usak.edu.tr

**Keywords:** action recognition, basketball training, inertial measurement unit (IMU), machine learning, wearable sensors

## Abstract

In this study, an action recognition system was developed to identify fundamental basketball movements using a single Inertial Measurement Unit (IMU) sensor embedded in a wearable vest. This study aims to enhance basketball training by providing a high-performance, low-cost solution that minimizes discomfort for athletes. Data were collected from 21 collegiate basketball players, and movements such as dribbling, passing, shooting, layup, and standing still were recorded. The collected IMU data underwent preprocessing and feature extraction, followed by the application of machine learning algorithms including KNN, decision tree, Random Forest, AdaBoost, and XGBoost. Among these, the XGBoost algorithm with a window size of 250 and a 75% overlap yielded the highest accuracy of 96.6%. The system demonstrated superior performance compared to other single-sensor systems, achieving an overall classification accuracy of 96.9%. This research contributes to the field by presenting a new dataset of basketball movements, comparing the effectiveness of various feature extraction and machine learning methods, and offering a scalable, efficient, and accurate action recognition system for basketball.

## 1. Introduction

Success in sports depends on mastering fundamental techniques that improve performance, reduce injury risk, and optimize training benefits [1]. Basketball technique represents all the specific movements performed by an athlete while playing the game, in accordance with the rules and tasks set by the team during a competition [2]. In basketball, dribbling, passing, shooting, and rebounding are the fundamental technical skills that enable players to play the game effectively and are often the focus of training. These movements are fundamental to both offensive and defensive play. Therefore, technical skills in basketball are considered one of the prerequisites for success [3].

Major sports events, like the Olympic Games and the NBA, significantly impact the popularity of basketball [4]. In their study, Kennedy and Dimick reported that 48% of college basketball players expect to become professional athletes [5]. For this reason, just like in all sports and different leagues, the goal in college basketball is to improve athlete performance. This can be achieved by combining the coach’s knowledge with data-driven scientific strategies, making training more personalized [6].

The wearable sensors can analyze and identify athletes’ movements, helping them master basketball skills and enhance their game performance to improve athlete performance in basketball [7,8]. Action recognition technologies using Inertial Measurement Unit (IMU) sensors significantly increase the performance of basketball players by providing quantitative analysis and guidance for shooting techniques [9]. Additionally, wearable sensors can monitor the athlete’s body posture and movement trajectory in real time, transmitting data to a mobile image processing system for analysis and processing [10]. IMUs assist in workload assessment and performance evaluation by offering precise data collection on body movements [11]. IMU sensors provide very useful and critical information in the field of sports as they can be used to detect acceleration, speed, and angular position changes and determine the forces generated/applied by providing mass information. This capability is essential for performance analysis, training optimization, and injury prevention, as it allows coaches to provide tailored feedback based on players’ movements. However, accurately classifying basketball activities poses challenges due to the overlap of movements and variability in playing styles, particularly when considering cultural differences, as highlighted by the Hang-Time HAR dataset, which includes data from 24 players across the USA and Germany during both structured drills and unstructured games [12]. This dataset is significant for evaluating machine learning algorithms, particularly deep learning architectures, as it enables researchers to assess generalization across different players using techniques like leave-one-subject-out (LOSO) cross-validation. These technologies enable the accurate recognition of various basketball movements, such as shooting stances and gestures, contributing to personalized training plans and improving training effectiveness [13,14]. Moreover, wearable sensors like IMUs can identify individuals based on unique movement patterns, such as walking, showcasing their potential for personalized feedback and skill development in sports like basketball [15]. Basketball players can benefit from IMU-based action recognition technologies by receiving detailed feedback on their shooting techniques, which can lead to enhanced skill development and overall performance on the court.

IMU sensors are utilized in basketball for activity recognition, capturing dynamic movements such as dribbling, shooting, and passing [9,12,16,17,18,19,20,21,22]. IMUs are sophisticated microelectromechanical sensors (MEMSs) that integrate accelerometers, gyroscopes, and, sometimes, magnetometers to measure motion and orientation in three-dimensional space.

This research aims to establish the Hang-Time HAR dataset as a benchmark for the human activity recognition community, paving the way for future studies on preprocessing algorithms and neural network architectures tailored for sports applications, especially in complex scenarios where activities overlap and vary in pace. IMU-based action recognition technology can recognize shooting movements, such as set shots, layups, jump shots, and tipping, with an accuracy of 98.0% and an overall recognition accuracy of 98.5% [9]. Additionally, IMU sensors can detect fundamental basketball movements like standing, walking, running, jumping, dribbling, set shots, and jump shots with an impressive accuracy of 98.9% by employing neural network algorithms [23]. Furthermore, a new method based on image feature extraction and machine learning can accurately classify and recognize basketball shooting movements, providing a scientific foundation for the development of modern basketball training [13]. These advancements in IMU-based technologies offer valuable insights for coaches and players, enhancing training, performance, skill development, and injury prevention in basketball. In various studies, some have used a single sensor [16,17,22], while others have employed multiple sensors [9,18,19,20,21]. These studies show that wearable devices are typically worn on the arms, body, or legs, focusing mainly on movements such as shooting, passing, dribbling, and blocking.

Hu et al. placed reflective markers on the bony landmarks of 10 college players and 10 novice basketball players according to the Full Body Plug-in Gait Model. They examined four basketball skills: jump shot, layup, passing the ball, and dribbling the ball. For classification, they used Dynamic Time Warping (DTW) and a basketball activity classification model based on body kinematic measurements, achieving accuracy, recall, and specificity values of 98.4%, 98.3%, and 99.4%, respectively [18].

Asmara et al. used a smartwatch device as a wearable to collect information on passing, dribbling, and shooting activities among basketball players. They found that the action recognition accuracy was 81.62% [20]. Liu and Zhang collected data from 100 basketball players in four postures: shooting, passing, dribbling, and catching [21]. In the study, two IMU sensors were worn by athletes to gather data. They extracted multidimensional action posture features from both time and frequency domains. Deep Q-Learning with Recurrent Neural Networks (DRQN) was used for posture recognition, resulting in an average accuracy of 99.3% for posture recognition.

Eggert et al. developed an IMU-based algorithm to recognize jump shots in the random basketball action sequences of 10 male basketball players [19]. The cross-validation for excluding one subject from the network showed values above 0.970 for recall and precision, with an area under the receiver operating characteristic curve of 0.995.

Hölzemann and Van Laerhoven aimed to track actions like dribbling, shooting, blocking, and passing during training and games using an IMU sensor in a smartwatch worn by three basketball players [16]. The highest overall accuracy performance of 87.5% was achieved with the Random Forest algorithm. They obtained 95% accuracy for specific actions like shooting, while, for some types of dribbling, the recall was only 44%.

Jiang and Zhang designed a hardware module to create a classification structure for basketball postures and collect data using inertial sensors [22]. Their study showed that a wearable device based on an improved algorithm achieved the highest accuracy of 99.4% in recognizing basketball sport postures.

Ross et al. used clustering-type machine learning algorithms, inputting segment linear accelerations and angular velocities to classify basketball players as elite or novice [17]. They included 542 athletes in the study for model validation and asked them to perform 21 unique movements to test their range of action, stability, strength, and balance. They assessed seven dynamic movements: drop jump, bird-dog, hop-down, lunge, step-down, L-hop, and T-balance for analysis. However, using metrics obtained through IMUs and Linear Discriminant Analysis (LDA), they accurately classified 75.1% to 84.7% of the athletes as elite or novice based on their movement. In another study using multiple sensors, an IMU sensor-based bracelet was used to collect shooting action data from 15 amateur and 5 elite basketball players, focusing on fixed shots, layups, jump shots, and tip-ins [9]. They achieved an accuracy of 98.0% in recognizing shooting actions across four categories, with an overall recognition accuracy of 98.5% for 18 different shooting movements.

In addition to the benefits provided, action recognition technologies using IMUs in basketball face various challenges. These include the need for effective hardware–software collaboration [24], the requirement for accurate posture definition and gesture recognition [13], reliance on the coach’s experience with traditional training methods [23], potential drift in speed and position vectors due to timing integration issues with IMU data [25], and the necessity for advanced spatial structure and temporal feature expression in skeletal sequences for better recognition performance [26].

Research shows that as the number of sensors increases, the performance metrics of machine learning improve; however, increasing the number of sensors also raises costs and processing intensity, while comfort may decrease. In basketball, the chest and back are considered the most suitable body areas for using IMUs due to their advantages in predicting ground reaction forces during vertical jumps [27]. IMUs placed on the sacrum have shown better performance in predicting ground reaction forces during jumps compared to those on the back and chest [27]. The lower risk of sensor mobility during the gesture minimizes motion artifacts and improves measurement reliability [28]. The reduced dependence on anatomical variations among subjects enhances the generalizability of results across diverse athletes [29]. Placing IMUs on the chest offers benefits for various applications, such as activity recognition, posture analysis, and cardiopulmonary parameter estimation [30]. In contrast, using IMUs on the hands, arms, or legs can create limitations. Additionally, IMU acceleration in lower extremities, such as the tibia and femur, can effectively measure peak acceleration during running, providing valuable insights into loading rates and impact peaks in runners [31]. Therefore, while chest and back placements are excellent for IMUs in basketball, other body regions may not offer the same level of accuracy and applicability.

This study aimed to develop a system capable of recognizing fundamental basketball movements by placing an IMU sensor on a vest-type wearable that would not cause discomfort for the wearer while playing basketball. The intended scientific contributions of this paper include the following:

- Presenting a dataset that includes previously undefined movements in the field of basketball.
- Comparing high-performance feature extraction and machine learning methods aimed at action recognition in basketball.
- Offering a high-performance, low-cost action recognition system for the basketball field with fewer sensors.
- Integrating explainable artificial intelligence (XAI) into an action recognition model.

The following sections will present the materials used, methods, findings, discussion, and conclusion in that order.

## 2. Materials and Methods

**Figure 1:**
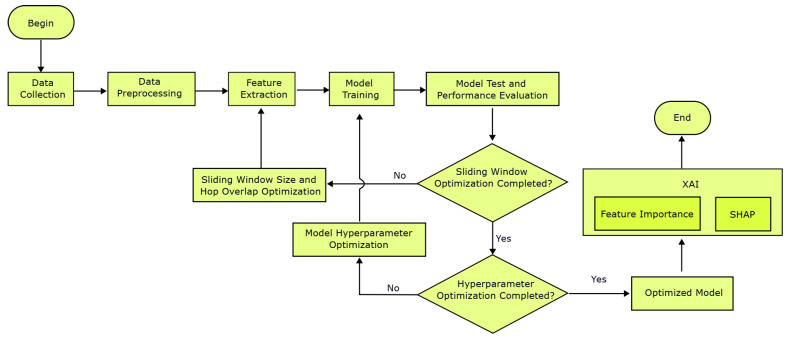
General flowchart.

In this study, an action recognition system for basketball movements was developed using machine learning. To achieve this, 3-axis accelerometers and 3-axis gyroscope sensors were used. Basketball players wearing a vest equipped with an IMU were involved in the data collection and action labeling process while performing relevant basketball skills. The materials used for data collection and labeling are presented in this paper in detail. The IMU signals underwent data preprocessing and feature extraction to prepare the dataset. The dataset was then subjected to machine learning, and the model with the highest performance was selected through performance evaluation before being deployed for use. The data collected from these sensors were processed using a specific classification algorithm to recognize certain basketball movements. The workflow for selecting the classification model is shown in Figure 1. After collecting and labeling the data, preprocessing and feature extraction steps were performed. Then, model training and evaluation were carried out. This process continued until the sliding window optimization and hyperparameter optimization for the chosen algorithm were complete. These optimization processes were performed using grid search, which involves testing a certain number of parameters and selecting the one that gives the best result. On the optimized model, explainable artificial intelligence was applied to understand how the model worked and enable more detailed studies in the future. In this stage, feature importance and Shapley Additive Explanations (SHAPs) were used.

### 2.1. Data Collection and Labeling

**Figure 2:**
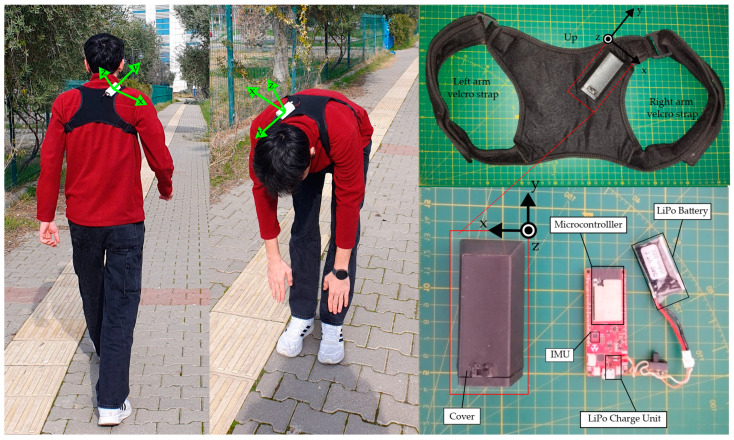
Back vest and IMU sensor.

In the data collection process, the wearable device shown in Figure 2 was placed on top of the vest. The wearable device consisted of a microcontroller (Deneyap Development Board (https://cdn.deneyapkart.org/media/upload/userFormUpload/NqjMEbvV2XUXk19JF68DzXA7H8Ok9Leo.pdf)). This microcontroller board consisted of a 240 MHz Dual Core Tensilica LX6 microprocessor (https://www.espressif.com/sites/default/files/documentation/esp32_datasheet_en.pdf), Bluetooth Low Energy, an IMU (LSM6DSM 3-Axis Gyroscope and 3-Axis Accelerometer), a charging unit, and a Li-Po battery (3.3 V). This board was placed inside a 3D-printed case and attached to a vest, and the accelerometer axes of the IMU are shown in Figure 2. IMU scale ranges of accelerometers were set to ±2 g and the gyroscopes were set to ±250 deg/s. The x-axis of IMU extended horizontally from the center of the sensor toward the right side of the wearer. The y-axis extended vertically upward, perpendicular to the ground and parallel to the spine of the athlete. The z-axis extended horizontally forward, perpendicular to the x-axis and aligned with the athlete’s forward-facing direction. The sensor’s coordinate system was maintained consistently across all subjects by securing the device in a fixed position within a rigid plastic casing, as shown in Figure 2.

**Figure 3:**
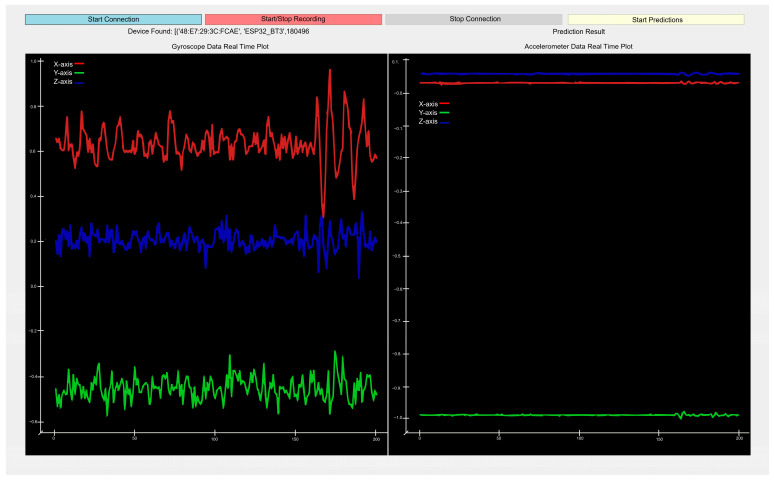
Data collection and labeling program GUI.

**Figure 4:**
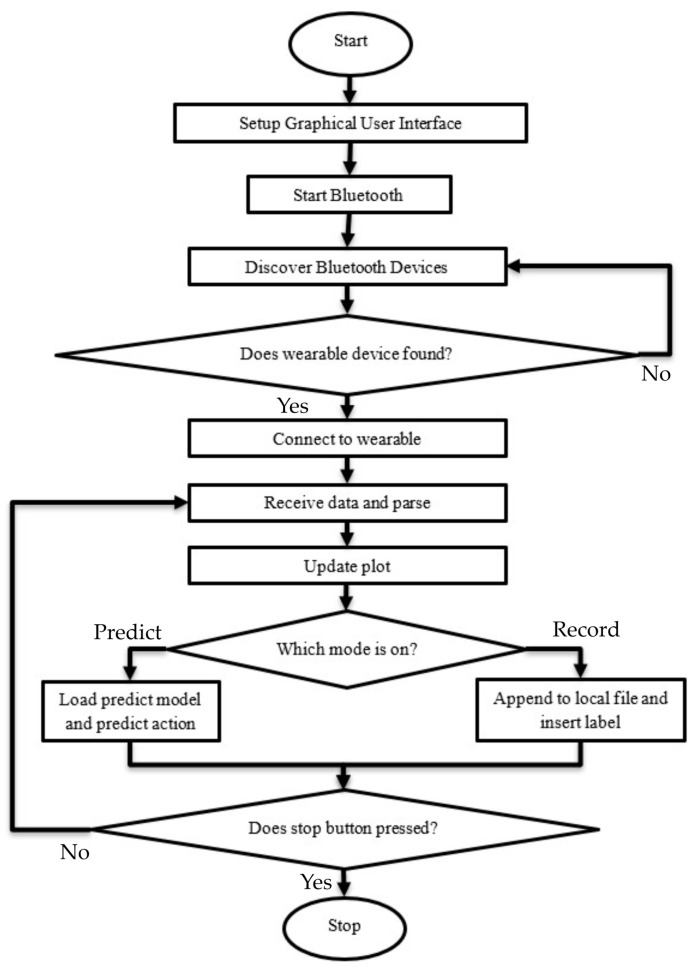
Data collection program flowchart.

Data collection and labeling were carried out using the data collection and labeling program shown in Figure 3. The cover was attached to the vest so that it always stayed in the same place. The circuit and battery could be removed from the plastic cover and maintenance could be performed if necessary. The same vest was worn by each user, but more than one vest of the same size was also produced for spare use. The flowchart of the program is presented in Figure 4. First, the graphical user interface (GUI) shown in Figure 3 was set up. Then, the computer’s Bluetooth module was activated. It scanned for nearby Bluetooth devices and connected to the wearable IMU device that had been previously paired with the program. If the device was not powered on or could not be found during the scan, it searched again. Once the connection was established, the data received from the device were time-stamped and parsed. Depending on the user’s request, the data could be used for labeling or predicting actions based on incoming data. The incoming data were displayed in real time on the drawing screen for each axis. In labeling mode, the action entered manually by the user was assigned as a file label. In prediction mode, after the trained model was introduced to the program, the action corresponding to the incoming data was predicted automatically. The program could be terminated at any time if the user wished to stop it.

**Figure 5:**
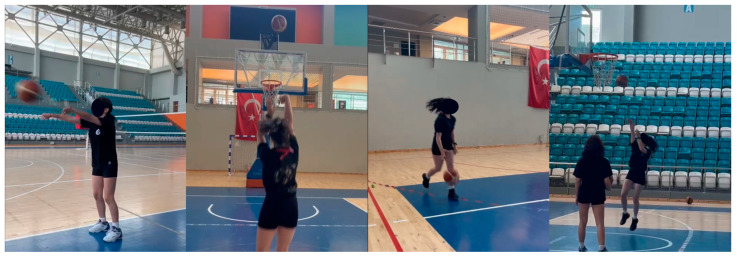
Sample basketball actions with wearable IMU.

Twenty-one basketball players (12 male and 9 female) aged between 19 and 24 years voluntarily participated in this study. The criteria for participation included having no medical conditions that would prevent completion of the tests, not performing any high-intensity exercise before the study, being right-handed, and being a member of the team. The research protocol followed the Declaration of Helsinki and was approved by the Uşak University Institutional Ethics Committee (decision no: 376-376-10, dated 2 May 2024). The players were clearly informed about the procedures to be applied. For each participant, personal information and height and weight measurements were taken and recorded. Then, they were asked to perform basketball-related actions, including dribbling, chest-passing, shooting, right-handed pivoting, and standing still. The standing still action involved the player wearing a sensor and remaining stationary. Examples of movements captured during data collection are shown in Figure 5. IMUs used for data collection operated at a sampling rate of 250 Hz and the data for each movement were recorded for a duration of 30 s. The number of repetitions of the movements varied according to the length of the movement. In this study, the repetitions were not so many that they would cause fatigue, but the focus was on variety.

### 2.2. Data Preprocessing and Feature Extraction

After data collection, preprocessing and feature extraction were carried out. During the movements of the basketball players, data from a 3-axis accelerometer and a 3-axis gyroscope were collected using IMU sensors. A sliding window technique was applied to each axis and each sensor. The collected data were recorded for 30 s for each movement. Data readings that were inaccurate were filtered, and normalization processes were applied. Using the sliding window method on the raw data, features such as the minimum, maximum, peak-to-peak, integral of simple squares, root mean square of squares, total of absolute differences, total of absolutes, skewness, and kurtosis were extracted, as presented in Table 1. According to the equations in Table 1, *x* represents the signal elements in the defined window; *n* is the last index of the window, which is the length of the window; *µ* is the mean; and *σ* is the standard deviation.

### 2.3. Model Training and Testing

The feature extraction process was carried out on a sample basis, not a subject basis. After feature extraction was performed for all subject data, the examples were combined to form a single dataset. The overlap value in the sliding window technique directly changed the number of samples extracted. Increasing the overlap value caused an increase in the intersecting data in the time series and the number of samples. Since the technique used in this study focused on selecting the optimum sliding window parameters, firstly, method selection was performed with k-fold cross-validation. Then, we used the Leave One Subject Out (LOSO) cross-validation method to rigorously evaluate the generalizability of the proposed action recognition model.

#### 2.3.1. K-Fold Cross-Validation and Model Selection

The dataset was divided into two parts: 75% for training and 25% for testing. For selecting the best model, learning models were evaluated on the training dataset using stratified 5-fold cross-validation. Test results were then applied separately to the test dataset after selecting the best model. The training data were divided into 5 equal parts while preserving the class distribution. Each part was used once as a test set, while the remaining four parts were used for training the model. After this process, the model with the best performance was selected. Finally, the selected model was tested on the test dataset, which helped to understand how well it performed on data it had never seen before. Classification models were created using algorithms including K-Nearest Neighbor (KNN), decision tree (DT), Random Forest (RF), Adaptive Boosting (AdaBoost), and Extreme Gradient Boosting (XGBoost). These models were trained to classify actions such as standing still, passing, shooting, dribbling, and jumping. The trained models were then tested on the test dataset, and their classification performance was evaluated.

KNN is a simple machine learning algorithm used for classification and regression. It works by finding the ‘k’ closest data points (neighbors) to a given input and making predictions based on these neighbors. The key idea is that similar data points will be close to each other. The main hyperparameters for KNN are ‘k’, the number of neighbors to consider, and the distance metric, such as Euclidean or Manhattan distance, which determines how the distance between points is calculated. Choosing the right ‘k’ value and distance metric is crucial for the algorithm’s performance [32,33].

The DT algorithm works by splitting data into smaller subsets based on certain conditions. It starts at the root node and divides the data at each node based on the feature that best separates the data into different classes or values. This process continues until the algorithm reaches a predefined depth or until it cannot split the data any further. Hyperparameters for decision trees include the maximum depth of the tree, the minimum number of samples required to split a node, and the minimum number of samples required to be at a leaf node. These hyperparameters help control the complexity of the tree and prevent overfitting. [34,35].

The RF algorithm is an ensemble learning method used for classification and regression tasks. It works by creating multiple decision trees during training and outputting the mode of the classes (classification) or the mean prediction (regression) of the individual trees. Key hyperparameters include the number of trees (n_estimators), the maximum depth of each tree (max_depth), and the number of features considered for splitting at each node (max_features). Adjusting these hyperparameters can help improve the model’s performance and prevent overfitting [36,37].

AdaBoost is a boosting algorithm that combines multiple weak learners to create a strong learner. It works by training a series of weak classifiers, typically decision trees, on different distributions of the training data. In each iteration, it adjusts the weights of incorrectly classified examples to focus on harder cases. The final model is a weighted sum of the individual weak learners. Key hyperparameters include the number of weak learners (n_estimators) and the learning rate, which controls the contribution of each weak learner [38,39].

The XGBoost is a powerful machine learning algorithm based on gradient boosting. It builds a series of decision trees, where each tree corrects the errors of the previous one. This method helps improve the accuracy of predictions. XGBoost uses several important hyperparameters to tune its performance, such as ‘learning_rate’, which controls how much each tree influences the final prediction; ‘n_estimators’, which determines the number of trees; and ‘max_depth’, which limits the depth of each tree to prevent overfitting. Properly tuning these hyperparameters is essential for obtaining the best results from the model [40].

#### 2.3.2. Leave One Subject out Cross-Validation

The generalizability of the proposed action recognition model was evaluated using the LOSO cross-validation method, which provides a subject-independent estimate of model performance, ensuring a model’s ability to generalize effectively to new users [41]. LOSO cross-validation in sports action recognition offers several significant benefits, particularly in enhancing the reliability and generalizability of models. This method addresses the inherent correlations in data collected from the same subjects, which can lead to the overestimation of model performance when traditional k-fold cross-validation is applied [42]. By ensuring that the training and testing datasets are independent, LOSO cross-validation provides a more accurate assessment of a model’s ability to be generalizable to unseen data. This approach involves iteratively using data from one subject as the test set, while the data from all other subjects are used for training the model. LOSO ensures that the model is tested on unseen data from individuals not included in the training set, making it particularly suitable for scenarios where inter-subject variability can significantly influence performance. The LOSO process was implemented as follows:

- **Data Partitioning:** For each iteration, data from one subject were completely withheld from the training set and used exclusively for testing. This process was repeated for all 21 subjects in the dataset, ensuring each subject served as the test set exactly once.
- **Model Training:** The selected learning algorithm was trained on the data from the remaining 20 subjects, utilizing the optimized hyperparameters identified in prior experiments.
- **Evaluation:** The trained model was then applied to the withheld subject’s data to generate predictions. Performance metrics such as accuracy, precision, recall, and F1-score were computed for each iteration.
- **Aggregation:** The final performance metrics were calculated by averaging the results across all iterations.

The inclusion of LOSO cross-validation provides a robust evaluation framework by simulating a real-world scenario where the model must perform on new, unseen individuals. This method inherently tests the model’s ability to generalize across different participants, accounting for variations in movement styles, physical attributes, and sensor placement inconsistencies.

### 2.4. Action Classification Performance Metrics

The performance metrics of the models obtained for action classification are presented in Table 2 [43,44]. For comparison, the main metric used for this study was accuracy. Accuracy is a metric that measures how well a model performs on all test data. It is calculated as the ratio of correctly classified examples to the total number of examples. In other words, it shows how accurate a model’s predictions are. Precision measures how many of the predictions made by a model for the positive class are actually true. In other words, it shows how accurate a model’s positive predictions are. Recall measures the rate at which a model correctly identifies positive examples. In other words, it shows how many actual positives are predicted correctly. The F1-score creates a balance between precision and recall. It is particularly useful for evaluating the overall performance of a model when there is a class imbalance. The F1-score is calculated as the harmonic mean of precision and recall.

### 2.5. Optimized Model Explanation

Explainable artificial intelligence (XAI) aims to enhance the transparency and interpretability of machine learning models by explaining how a model works. In this context, this study employed feature importance and Shapley Additive Explanation (SHAP) methods. Feature importance helps identify which features significantly impact model predictions, aiding in model improvement and decision-making [45]. A common method is to measure how performance metrics change by permuting the features to determine their importance [46]. One of the most popular methods involves selecting accuracy as the performance metric and observing the decrease in accuracy within the model. This method ensures a clear understanding of feature importance by maximizing the contribution of the remaining features to a model’s performance [47,48]. SHAP aims to explain a model by addressing the “black box” nature of machine learning models, assigning importance scores to features based on their contributions to model predictions [49]. SHAP values are calculated using game theory principles, where each feature’s contribution is evaluated considering all possible combinations of features [50]. SHAP calculates the contribution of each feature by considering all possible combinations of features and their interactions. For a given prediction, SHAP values are computed by evaluating the change in the expected prediction when a feature is included versus when it is excluded. The basic steps of SHAP can be summarized as follows [51,52]:

- **Model Training:** A machine learning model is trained on the dataset, capturing the relationships between input features and the output.
- **Shapley Value Calculation:** For each prediction, SHAP computes Shapley values, which quantify the contribution of each feature by considering all possible combinations of features. The Shapley value *ϕ*_i_ for a feature i is calculated using Equation (1). In this equation, N is the set of all features, S is a subset of features that does not include feature i, and f(S) is the model’s prediction when only the features in subset S are included. f(S∪{i}) is the prediction when feature I is added to subset S.

(1) $$\phi_{i}=\sum_{S\subseteq N\setminus \{i\}}\frac{\left|S\right|!\cdot \left(\left|N\right|-\left|S\right|-1\right)!}{\left|N\right|!}\;\cdot \;\left(f\left|S\;\cup \{i\}\right|-f(S)\right)$$

- **Feature Attribution:** The calculated Shapley values are used to assign importance scores to each feature, indicating their influence on the model’s output.
- **Visualization:** The results can be visualized using various plots to facilitate the understanding of feature impacts on predictions.

## 3. Results and Discussion

### 3.1. Model Selection with Sliding Window and Hyperparameter Optimization

In this study, we first tested the KNN, decision tree, Random Forest, AdaBoost, and XGBoost algorithms using various window sizes [100, 150, 200, 250] and hop overlap percentages [75, 50, 25, 0]. The accuracy values of the machine learning algorithms based on sliding window width and hop overlap percentages are presented in Table 3. According to the results, the highest performance was achieved using the XGBoost algorithm with a window size of 250 and a hop overlap percentage of 75%, yielding an accuracy value of 0.966. Examining Table 3, we can observe that as the window size increased and the overlap percentage rose, the accuracy value also increased.

After model selection and sliding window optimization, the chosen algorithm was XGBoost, with a sliding window width of 250 and an overlap value of 75%. For these parameters, the number of estimators for the XGBoost algorithm was [100, 150, 200], the learning rate was [0.1, 0.05, 0.01], the maximum depth was [3, 4, 5], the minimum child weight was [1, 2, 3], the subsample rate was [0.6, 0.7, 0.8], and the subsample ratio of columns was [0.6, 0.7, 0.8]. Within the scope of optimization of the hyperparameters of the XGBoost algorithm, the changes in accuracy values obtained for different parameter settings are summarized in Table 4 (Q1: First Quartile (25th percentile), Q3: Third Quartile (75th percentile)). The results obtained during the hyperparameter optimization of the XGBoost algorithm allowed for the identification of key hyperparameters that significantly impacted the model’s accuracy. Increasing the number of estimators from 100 to 200 led to a noticeable improvement in accuracy, with the median value rising from 0.941 to 0.955. Similarly, increasing the learning rate from 0.01 to 0.1 enhanced accuracy by 4.36%, raising it from 0.919 to 0.959. When the maximum depth parameter was increased from 3 to 5, the median accuracy also improved, reaching 0.955 from 0.941. The optimization of other parameters, such as the minimum child weight and subsample ratio, resulted in the best performance of the model, with 200 estimators, a learning rate of 0.1, a maximum depth of 5, a minimum child weight of 2, and a subsample ratio of 0.6. With these settings, the model achieved an accuracy of 0.969, precision of 0.970, recall of 0.969, and F1 score of 0.969.

To evaluate the generalizability of the proposed model, LOSO cross-validation was performed, where the data from one subject were entirely left out for testing while the model was trained on the remaining subjects. This process was repeated for all subjects, ensuring that each individual contributed to the testing phase. The results obtained using the optimized XGBoost algorithm with LOSO cross-validation demonstrated robust performance, achieving an accuracy of 0.878, an F1-score of 0.878, precision of 0.879, and recall of 0.841. These metrics confirmed the model’s ability to generalize well across different participants, even in the presence of inter-subject variability. While the LOSO results were slightly lower compared to those achieved using stratified cross-validation, the slight decrease in performance was expected due to the more stringent nature of LOSO, which simulates real-world variability. This validation highlights the robustness of the model and its potential applicability in practical settings, where the system may encounter data from unseen individuals. Additionally, the consistency of the performance metrics across subjects underscores the reliability of the model in recognizing basketball movements using a single wearable IMU sensor.

**Figure 6:**
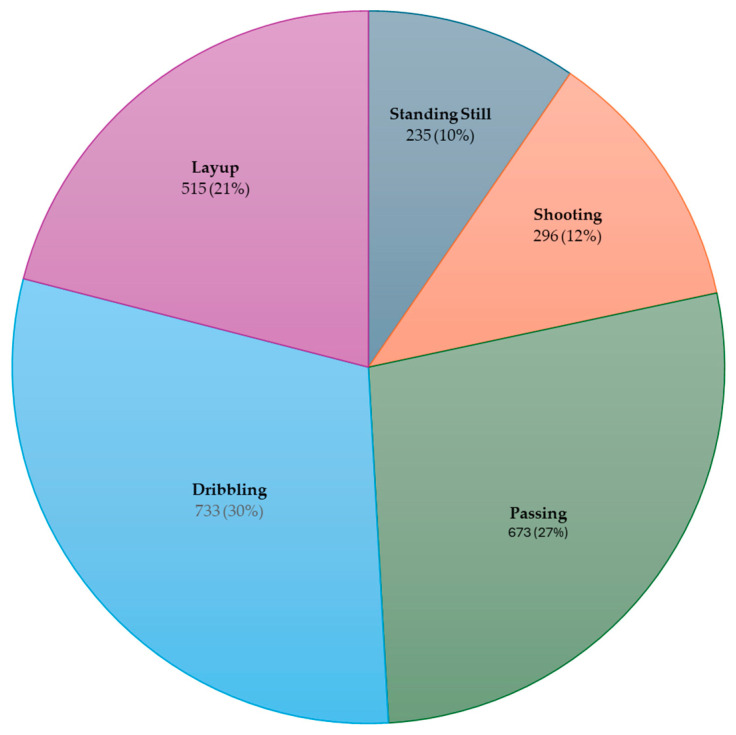
Distribution of dataset by number of samples.

**Figure 7:**
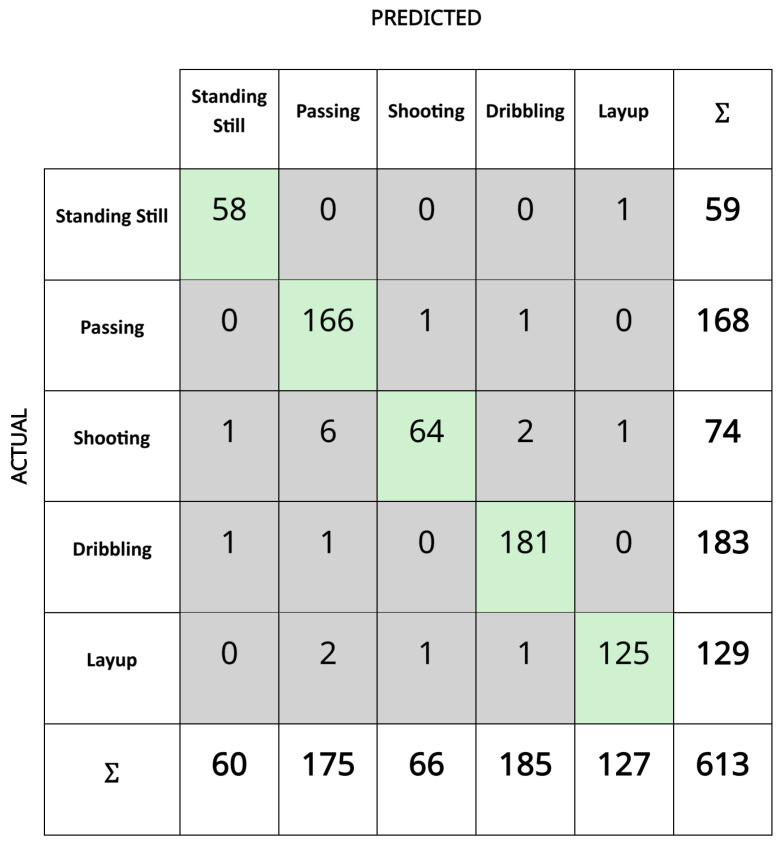
Confusion matrix of XGBoost for test dataset.

**Figure 8:**
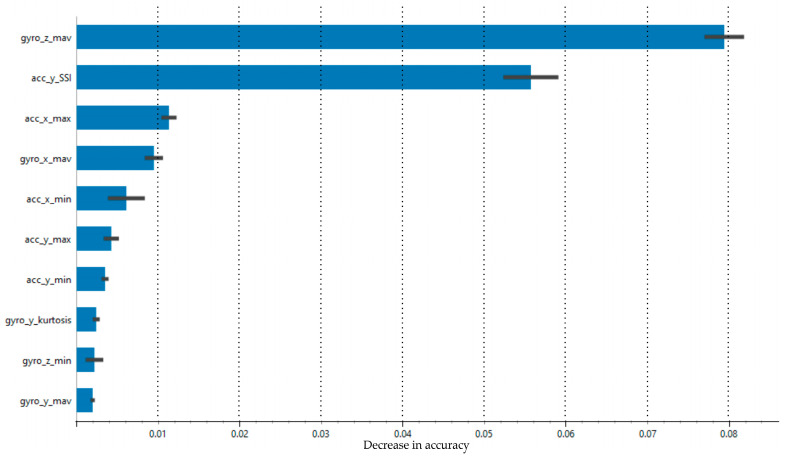
Feature importance in action classification with XGBoost.

The distribution of the outputs in the final dataset obtained after feature extraction according to the number of samples is presented in Figure 6. Accordingly, there were standing still (235 samples), passing (673 samples), shooting (296 samples), dribbling (733 samples), and layup (515 samples) classes with a total of 2452 samples. When the entire dataset was divided into 75% training and 25% testing data, 1839 samples were assigned training and 613 samples were assigned to testing. According to the confusion matrix analysis, which is shown in Figure 7, the model exhibited high accuracy and consistency, with a very low rate of misclassification. These results demonstrate that the XGBoost algorithm could be significantly optimized with the correct hyperparameter settings. Figure 8 presents the feature importance ranking for the XGBoost machine learning model used in the action recognition system developed for basketball movements. The figure shows the contribution of each feature to the accuracy of the classification model. Accordingly, the mean absolute of the gyroscope’s z-axis (gyro_z_mav), the simple square integral (SSI) of the y-axis accelerometer (acc_y_SSI), and the maximum of the accelerometer’s x-axis (acc_x_max) were the features that had the greatest effect on the accuracy value. The dominance of the gyroscope and accelerometer features suggests that movement data from both sensors were crucial for accurate basketball action classification. Movements along the z-axis seemed particularly critical, especially for actions like dribbling and shooting, which involve vertical and rotational movements.

**Figure 9:**
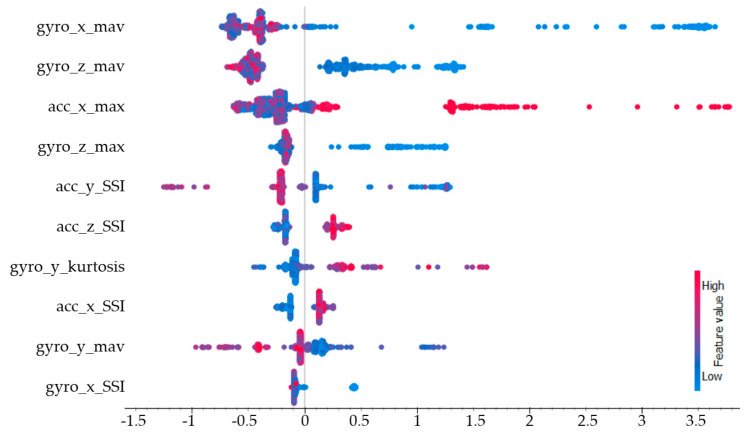
SHAP values for the standing still class.

In the explanation of the model’s performance, SHAP values were calculated for each class. The SHAP values calculated for standing still are presented in Figure 9. The three features with the highest impact for the standing still action were the mean absolute of the gyroscope’s x-axis (gyro_x_mav), the mean absolute of the gyroscope’s z-axis (gyro_z_mav), and the maximum of the accelerometer’s x-axis (acc_x_max). Since a stationary posture was maintained during this movement, the gyroscope sensor data were more prominent.

**Figure 10:**
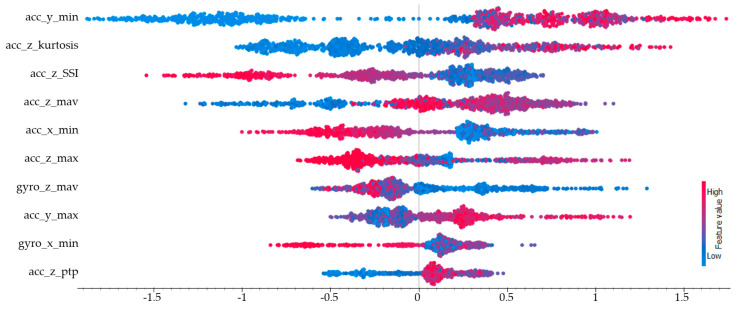
SHAP values for the passing class.

The SHAP values calculated for the passing class are presented in Figure 10. The three features with the highest impact were, respectively, the minimum value of the y-axis accelerometer (acc_y_min), the kurtosis value of the z-axis accelerometer (acc_z_kurtosis), and the simple square integral (SSI) of the z-axis accelerometer (acc_z_SSI). Five of the ten most important features in the classification of the passing action were derived from the z-axis of the accelerometer. This indicates that the z-axis movements of the action had high discriminative power. Although this action was performed while stationary, it involved some mobility, which is why the accelerometer features exhibited higher impact values.

**Figure 11:**
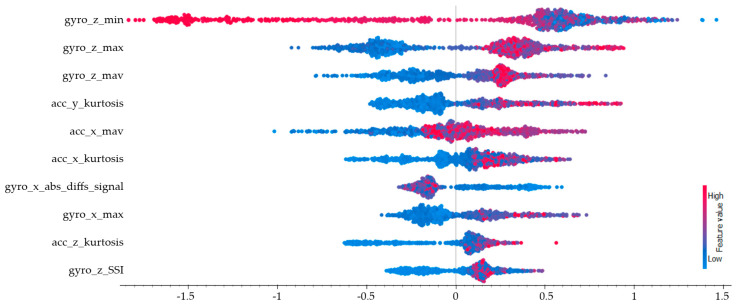
SHAP values for the shooting class.

The SHAP values calculated for the shooting class are presented in Figure 11, with the top three features with the highest impact being the minimum value of the z-axis gyroscope (gyro_z_min), the maximum value of the z-axis gyroscope (gyro_z_max), and the mean absolute value of the z-axis gyroscope (gyro_z_mav), respectively. Due to the movement along the z-axis during the shooting action, the gyroscope values on the z-axis showed high impact values. Since this action involves posture/orientation and aiming processes, it was observed that the gyroscope-related features had a higher impact. The shooting class showed a lower hit rate compared to other basketball movements like dribbling and passing. This difference might have been because shooting is a more complex action that requires careful control of body movements and balance, making it harder to perform consistently. The data showed that the way players moved during shooting, especially their hand and body angles, varied a lot between individuals. Different shooting styles among players could also be a reason for the lower accuracy. To improve results for shooting, using extra sensors on the arms or wrists might help capture the movement better. Adjusting how the data are processed could also make the system more accurate in recognizing shooting actions.

**Figure 12:**
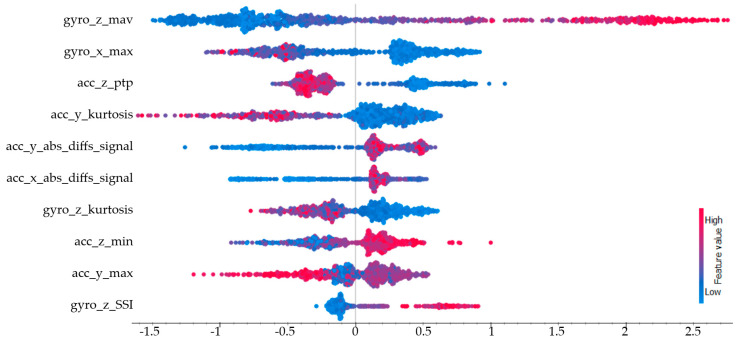
SHAP values for the dribbling class.

The SHAP values calculated for the dribbling class are presented in Figure 12. The three features with the highest impact were, respectively, the mean absolute value of the gyroscope on the z-axis (gyro_z_mav), the maximum value of the gyroscope on the x-axis (gyro_x_max), and the peak-to-peak value of the accelerometer on the z-axis (acc_z_ptp). Since dribbling involves movements in various directions, no axis could be said to be dominant during this action. Both gyroscope and accelerometer features showed diversity.

**Figure 13:**
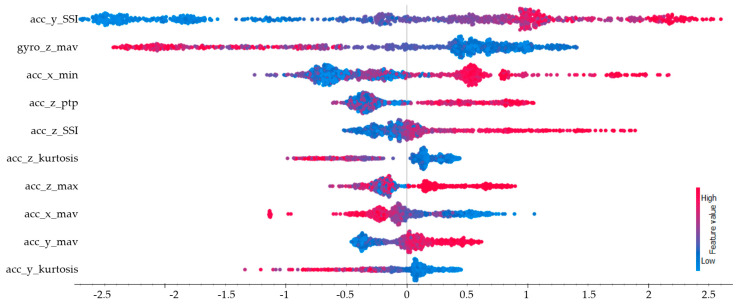
SHAP values for the layup class.

The SHAP values calculated for the layup class are presented in Figure 13. The features with the highest impact for this action were, in order, the y-axis accelerometer simple square integral (acc_y_SSI), the z-axis gyroscope mean absolute value (gyro_z_mav), and the x-axis gyroscope minimum value (gyro_x_min). In the layup action, the dribbling, shooting, and layup actions are combined, so similar features showed a high impact in this action as well. The z-axis gyroscope maximum value was among the top three features with the highest impact in the layup, dribbling, and shooting actions. Similarly, the kurtosis value of the y-axis accelerometer was also among the top ten most effective features for these three actions. The x-axis accelerometer mean absolute value (acc_x_mav) and the kurtosis value of the z-axis accelerometer were common influential features in both shooting and layup.

### 3.2. Comparison with Related Works

In this paper, the proposed method and other studies in the field of basketball are summarized in Table 5. Action recognition technologies that use IMUs in basketball face various challenges. These include the need for effective hardware–software collaboration [24], the requirement for accurate posture definition and gesture recognition [13], reliance on the coach’s experience with traditional training methods [23], potential drift in speed and position vectors due to issues with the time integration of IMU data [25], and the necessity for advanced spatial structures and temporal feature expressions in skeleton sequences to improve recognition performance [26]. IMU-based technologies improve basketball training by enhancing gesture recognition and posture analysis. While adding sensors improves accuracy, it may also increase costs and reduce comfort. In basketball, the chest and back are the most suitable body areas for using IMUs, particularly because of their advantages in predicting ground reaction forces during vertical jumps [27]. Placing IMUs on the chest offers benefits for various applications, such as activity recognition, posture analysis, and predicting cardiopulmonary parameters [30]. Back-mounted sensors can be integrated into clothing, making them less intrusive compared to limb sensors, which may restrict movement [53]. Studies show that back-mounted IMUs can yield reliable data for various activities, including vertical jumps, with high correlation coefficients, indicating their effectiveness in performance analysis [54]. On the other hand, using IMUs on the hands, arms, or legs may present limitations. For instance, IMUs placed on the sacrum have shown better performance in predicting ground reaction forces during jumps compared to those on the back or chest [27]. Additionally, the acceleration of IMUs positioned on the lower extremities, like the tibia and femur, can effectively measure peak acceleration during running and provide valuable insights into loading rates and impact peaks for runners [31]. Therefore, while the chest and back placements are ideal for IMUs in basketball, other body regions may not offer the same level of accuracy and applicability. Hu et al. used Dynamic Time Warping (DTW) as a classifier based on body kinematic measurements for classifying basketball activities [18]. The accuracy, recall, and specificity achieved by this system were high, but it used 39 sensors. Thirty-nine reflective markers were placed on bony landmarks according to the Full Body Plug-in Gait Model and measured movements like jump shots, passes, and dribbling. Asmara et al. used a smartwatch device to gather information on passing, dribbling, and shooting activities in basketball [20]. However, the average action recognition accuracy was low, and only two sensors were used. In a study conducted by Liu and Zhang, athletes were equipped with two IMU sensors for data collection [21]. Features of multidimensional action postures were extracted from both time and frequency domains. In the study, Deep Q-Learning with Recurrent Neural Networks (DRQN) was used for posture recognition, demonstrating a high average accuracy for this task. However, the study also relied on multiple sensors. An IMU-based algorithm developed by Eggert et al. aimed to recognize jump shots in random sequences of basketball movements [19]. This approach showed high values for cross-validation, recall, and precision, with a high area under the curve for receiver operating characteristics. Yet again, the study used four sensors placed on the foot, lower back, upper back, and shooting hand. Hölzemann and Van Laerhoven’s study involved basketball players wearing a smartwatch with an IMU sensor on their wrist, aiming to track actions like dribbling, shooting, blocking, or passing during training and games [16]. They achieved a top overall accuracy of 87.5%. While high accuracy was attained for specific actions like shooting, lower recall was observed for some types of dribbling. In the module proposed by Jiang and Zhang, a wearable device based on an improved algorithm had the highest accuracy for posture recognition in basketball [22]. However, the study focused only on basketball stances. Ross et al. conducted a study with a single sensor and included 542 athletes [17]. Based on the action, metrics obtained using IMUs and Linear Discriminant Analysis (LDA) accurately classified 75.1% to 84.7% of athletes as elite or novice. Lian et al. included players using an IMU sensor-based wristband [9]. Although they achieved high accuracy in recognizing shooting movements across four categories, such as fixed shots, layups, jump shots, and free throws, the study focused solely on shooting skills and used five sensors. This study differs from existing research by designing a system that recognizes basketball actions with high performance, placing the IMU sensor in the less intrusive area of the lower back during games or training. With just one IMU sensor, it can classify a total of five different basketball movements with an impressive accuracy of 96.9%, surpassing other single-sensor systems.

## 4. Conclusions and Future Works

In this study, we developed a highly accurate movement recognition system using a single IMU sensor placed in a vest to recognize the movements of basketball players. The system successfully classified five different basic basketball movements with a high accuracy of 96.9%, thanks to the IMU sensor located on the back. This was achieved without causing discomfort during basketball training or matches, and it outperformed other single-sensor systems. This result shows that low-cost and user-friendly systems can also provide high performance in sports like basketball.

One of the key contributions of this study is the introduction of a new dataset to the existing literature and the comparative analysis of different movement recognition algorithms in the context of basketball. Additionally, developing a system that performs with high accuracy using fewer sensors serves as an important reference for future research. The system stands out as a significant tool for preparing individual training programs. Athletes can receive real-time feedback on their movements, which can help them improve their sports performance. At the same time, this system acts as a functional support tool for coaches, reducing their workload and minimizing the impact of subjective judgments. This enables athletes to be guided in a more scientific and data-driven manner.

While the proposed action recognition system using a single IMU sensor achieved high accuracy in classifying basketball movements, there are certain limitations to consider. The system was evaluated using a single sensor placed on the back, which, while effective, may not capture the full range of complex movements involving the arms and legs, such as shooting or passing variations with greater biomechanical complexity. Additionally, the dataset used, though diverse, included a limited number of participants, which may have reduced the generalizability of the results to larger and more diverse athletic populations. This study also focused on fundamental basketball movements, and the system’s performance in more complex sequences or game scenarios with increased movement overlap was not fully tested. Future research should explore the use of multiple IMU sensors placed on various body regions to capture a broader range of motion and improve the recognition accuracy for dynamic actions. Moreover, expanding the dataset with athletes of varying skill levels and physical characteristics will enhance the model’s robustness and generalizability. Incorporating physiological sensors, such as heart rate monitors or electromyography (EMG), could provide additional context for movement analysis and performance evaluation. Finally, leveraging deep learning techniques and real-time feedback mechanisms may further improve the system’s accuracy and usability for both coaches and players during live training and competitive scenarios.

## Figures and Tables

**Table 1:** Features and equations.

Feature Name	Variable Abbreviation	Equation
Minimum	min	Min(x) = min(x_1_, x_2_, …x_n_)
Maximum	max	Max(x) = max(x_1_, x_2_, …x_n_)
Peak-to-Peak	ptp	PP(x) = max(x_1_, x_2_,… x_n_) − min(x_1_, x_2_, …x_n_)
Simple Square Integral	SSI	$SSI(x)=\sum_{i=0}^{n}x_{i}^{2}$
Root Mean Square	rms	$RMS\left(x\right)=\sqrt{\frac{1}{n}\sum_{i=1}^{n}x_{i}^{2}}$
Absolute Differences	abs_diffs_signal	$AD\left(x\right)=\sum_{i=2}^{n}\left|x_{i}-x_{i-1}\right|$
Mean Absolute	mav	$AM\left(x\right)=\frac{1}{n}\sum_{i=1}^{n}\left|x_{i}\right|$
Skewness	skewness	$S\left(x\right)=\frac{1}{n}\sum_{i=1}^{n}{\left(\frac{x_{i}-µ}{\sigma }\right)}^{3}$
Kurtosis	kurtosis	$K\left(x\right)=\frac{1}{n}\sum_{i=1}^{n}{\left(\frac{x_{i}-µ}{\sigma }\right)}^{4}-3$

**Table 2:** Performance metrics.

Metric	Equation
Accuracy	$A=\frac{Number\;of\;True\;Classified\;Samples}{Total\;Number\;of\;Samples}$
Precision	$P=\frac{True\;Positives}{True\;Positives+False\;Positives}$
Recall	$R=\frac{True\;Positives}{True\;Positives+False\;Negatives}$
F1-Score	$F1=2\times \frac{P\times R}{P+R}$

**Table 3:** Accuracy of machine learning algorithms according to sliding window size and hop overlap values.

Window Size	Window Hop Overlap Percentage	KNN	DT	RF	AdaBoost	XGBosst
100	75	0.629	0.857	0.931	0.411	0.957
100	50	0.597	0.774	0.858	0.432	0.877
100	25	0.591	0.771	0.854	0.521	0.878
100	0	0.601	0.764	0.899	0.448	0.896
150	75	0.688	0.872	0.932	0.435	0.965
150	50	0.633	0.815	0.901	0.529	0.93
150	25	0.593	0.802	0.837	0.663	0.864
150	0	0.62	0.755	0.844	0.484	0.865
200	75	0.675	0.873	0.924	0.483	0.958
200	50	0.688	0.840	0.948	0.573	0.962
200	25	0.609	0.823	0.891	0.484	0.88
200	0	0.639	0.722	0.875	0.306	0.882
250	75	0.727	0.897	0.957	0.492	0.966
250	50	0.701	0.879	0.922	0.554	0.935
250	25	0.61	0.838	0.922	0.519	0.922
250	0	0.647	0.828	0.879	0.517	0.914

**Table 4:** Accuracy change in XGBoost hyperparameters.

Hyperparameter	Value	Median	Q1	Q3
Number of Estimators	100	0.941	0.925	0.949
	150	0.951	0.933	0.957
	200	0.955	0.935	0.961
Learning Rate	0.01	0.919	0.902	0.929
	0.05	0.947	0.943	0.955
	0.1	0.959	0.955	0.961
Maximum Depth	3	0.941	0.902	0.949
	4	0.949	0.921	0.957
	5	0.955	0.937	0.959
Minimum Child Weight	1	0.947	0.929	0.959
	2	0.945	0.931	0.957
	3	0.945	0.927	0.955
Subsample	0.6	0.945	0.925	0.957
	0.7	0.947	0.931	0.959
	0.8	0.945	0.929	0.957

**Table 5:** Action recognition in basketball.

Reference	# Sensor(s)	Wearable Placement	# Subject(s)	Action Classes	Performance
[9]	5	back, lower legs, feet	15	Player Identity, Shot Types	Accuracy: 0.985
[16]	1	wrist	3	Dribbling, Shooting, Blocking, Passing	Accuracy: 0.875
[17]	1	wrist, foot, waist	542	Player Level Classification	Accuracy: 0.847
[18]	39	upper body	20	Dribbling, Shooting, Passing, Layup	Precision: 0.984 Recall: 0.983 Specificity: 0.994
[19]	4	foot, lower back, upper back, hand	10	Jump Shooting, Non-Jump Shooting	Recall: 0.975 Precision: 0.980
[20]	2	wrists	NA	Dribbling, High Jump Shot	Accuracy: 0.816
[21]	2	wrist, foot, waist	100	Shooting, Passing, Dribbling, Catching	Accuracy: 0.993
[12]	1	wrist	24	Dribbling, Shooting, Passing, Layup, Rebound, Walking, Running, Standing, Sitting	F1-score: 0.24
[22]	2	upper limbs, lower limbs	10	Dribbling, Shooting, Passing, Jumping, Catching, Walking Running, Standing Still	Accuracy: 0.994
[23]	1	wrist	1	Dribbling (walking, running, in-situ), Shooting, Passing, Layup, Walking, Running, Jumping, Standing Still	Accuracy: 0.989
Proposed System	1	back	21	Dribbling, Shooting, Passing, Layup, Standing Still	Accuracy: 0.969

## Data Availability

The raw data supporting the conclusions of this article will be made available by the authors upon request and Supplementary Materials.

## Supplementary Materials

The following supporting information can be downloaded at: https://www.mdpi.com/article/10.3390/s25020563/s1.
